# A randomized controlled trial of brain training with non-action video games in older adults: results of the 3-month follow-up

**DOI:** 10.3389/fnagi.2015.00045

**Published:** 2015-04-14

**Authors:** Soledad Ballesteros, Julia Mayas, Antonio Prieto, Pilar Toril, Carmen Pita, Ponce de León Laura, José M. Reales, John A. Waterworth

**Affiliations:** ^1^Studies on Aging and Neurodegenerative Diseases Research Group, Universidad Nacional de Educación a DistanciaMadrid, Spain; ^2^Department of Informatics, Umea UniversityUmea, Sweden

**Keywords:** brain plasticity, cognitive aging, clinical trial, non-action video games, wellbeing

## Abstract

This randomized controlled study (ClinicalTrials.gov NCT02007616) investigated the maintenance of training effects of 20 1-hr non-action video game training sessions with selected games from a commercial package on several age-declining cognitive functions and subjective wellbeing after a 3-month no-contact period. Two groups of cognitively normal older adults participated in both the post-training (posttest) and the present follow-up study, the experimental group who received training and the control group who attended several meetings with the research team during the study but did not receive training. Groups were similar at baseline on demographics, vocabulary, global cognition, and depression status. Significant improvements in the trained group, and no variation in the control group had been previously found at posttest, in processing speed, attention and visual recognition memory, as well as in two dimensions of subjective wellbeing. In the current study, improvement from baseline to 3 months follow-up was found only in wellbeing (*Affection* and *Assertivity* dimensions) in the trained group whereas there was no change in the control group. Previous significant improvements in processing speed, attention and spatial memory become non-significant after the 3-month interval. Training older adults with non-action video games enhanced aspects of cognition just after training but this effect disappeared after a 3-month no-contact follow-up period. Cognitive plasticity can be induced in older adults by training, but to maintain the benefits periodic boosting sessions would be necessary.

## Introduction

The increasing longevity and the physical and mental declines that occur with aging motivate current research to investigate new avenues to promote or maintain healthy aging and independent living, delaying as much as possible cognitive and brain deterioration (Hertzog et al., 2009; Mozolic et al., 2011; Buitenweg et al., 2012). Age-related brain changes occurring in the prefrontal cortex and the medial temporal lobe system, including the hippocampus and the cerebellum (Raz et al., 2005; see Park and Reuter-Lorenz, 2009; Reuter-Lorenz and Park, 2014), are associated with declines in several cognitive processes including processing speed, working memory, executive functions and episodic memory (e.g., Salthouse, 1996; Baltes and Lindenberger, 1997; Nilsson, 2003; Hoyer and Verhaeghen, 2006). Other cognitive functions such as general knowledge, verbal abilities (e.g., Park et al., 2002; Hedden and Gabrieli, 2004; Craik and Bialystok, 2006), and implicit memory are mostly preserved in normal aging (e.g., Mitchell and Bruss, 2003; Wiggs et al., 2006; Ballesteros et al., 2008, 2009, 2013c). This latter type of memory is also preserved in mildly cognitively impaired older adults (Ballesteros et al., 2013b) and in Alzheimer's disease patients (Gabrieli, 1998; Ballesteros and Reales, 2004) despite great deterioration in explicit (episodic) memory. The preservation of implicit memory with aging is accompanied by altered neural priming, as shown in electrophysiological (Osorio et al., 2010; Sebastián and Ballesteros, 2012) and event-related functional magnetic resonance imaging priming studies, possibly as a form of compensatory neural activity (Ballesteros et al., 2013a).

The association of increased life expectancy and the occurrence of neurodegenerative diseases (e.g., Brookmeyer et al., 2011; Reitz et al., 2011) has stimulated interest in identifying factors that might protect older adults from cognitive decline and dementia. Thus, the interest in interventions that can improve and/or preserve cognitive and brain health in older adults has grown noticeably in the last decade (e.g., Smith et al., 2009; Hampstead et al., 2012; Anguera et al., 2013). Many studies support the potential for positive changes in older adults (e.g., see Hertzog et al., 2009; Valenzuela and Sachdev, 2009; Reuter-Lorenz and Park, 2014) due to neuroplasticity, understood as the ability of the brain to adapt to environmental change by modifying neural connectivity and brain function (Knaepen et al., 2010). Neuroplasticity was first shown in animal studies, suggesting experience-induced increases in the hippocampus of those individuals living in an enriched environment (e.g., Kempermann et al., 2002, 2004). Human studies have also shown neural plasticity at several levels of the neural system (e.g., Pascual-Leone et al., 2005; Raz et al., 2005) although larger in young adults than in older adults (e.g., Bialystok and Craik, 2006; Lee et al., 2008). The prolonged mismatch between functional organismic supplies and environmental demands produces cognitive plasticity and indicates the capacity of the brain for implementing behavioral flexibility (Lövdén et al., 2010; Bavelier et al., 2012; Park and Bischof, 2013). Training programs have been effective in improving older adults' cognitive performance in memory tasks (e.g., Craik et al., 2007; Smith et al., 2009; Hampstead et al., 2012) and other functions such as attention, working memory, reasoning, speed of processing, cognitive control and dual-task switching (e.g., Edwards et al., 2005; Bherer et al., 2006; Erickson et al., 2007; Karbach and Kray, 2009; Berry et al., 2010; Mozolic et al., 2011, for a recent meta-analysis of executive-control and working memory training in older adults, see Karbach and Verhaeghen, 2014).

Several recent training studies have reported positive effects in different aspects of older adults' cognition after training with video games (e.g., Basak et al., 2008; Buschkuehl et al., 2008; Nouchi et al., 2012; Anguera et al., 2013; Ballesteros et al., 2014). However, other studies did not find significant transfer effects of training to measures of cognitive functioning (e.g., Ackerman et al., 2010; Owen et al., 2010; Boot et al., 2013a; Reddick et al., 2013). A systematic review (Kueider et al., 2012) and three recent meta-analytic studies (Powers et al., 2013; Lampit et al., 2014; Toril et al., 2014) have examined the effectiveness of computer-based interventions in cognitively healthy older adults. Toril et al. (2014) found that training healthy older adults with video games enhanced cognitive functioning. Twenty experimental studies published between 1986 and 2013 were included in the meta-analysis. The studies included were video game training interventions with pre-and post-training outcomes. The results of the meta-analysis indicated that video game training produced positive effects on several cognitive functions, including reaction time, attention, memory, and global cognition. Positive effects were moderated by variables such as the age of the trainees and the length of the training program, suggesting that the benefits of training increase as participants get older and that training effects are greater when training is short (1–6 weeks) than when it is long (7–12 weeks), perhaps because long training schedules may cause older adults to feel bored. The mean effect size was 0.37 (ET = 0.05) with a 95% confidence interval of between 0.26 and 0.48, which is considered moderate. These results suggest neurocognitive plasticity in the aging human brain, as training with video games enhanced cognitive performance on several untrained functions.

We have previously reported the post-training results of this randomized controlled trial conducted to investigate the effects of training older adults with non-action video games to determine whether the training benefits transfer to a broad number of cognitive functions (Ballesteros et al., 2014; Mayas et al., 2014). In this longitudinal study, a group of healthy older adults were trained in the laboratory for 20 1-h sessions over the course of 10–12 weeks. In each session, the trainees practiced 10 non-action video games twice. We compared their pretest and posttest results on a series of psychological tests and computerized experimental tasks with those of a control group to examine possible transfer of training to untrained tasks. The main question was whether the trained older adults would improve after training several cognitive functions that decline with age as well as improve in subjective wellbeing as compared to a control group. The post-training results of this randomized controlled trial showed significant improvements in the trained group, and no change in the control group, on choice reaction time (processing speed), immediate and delayed visual recognition memory, as well as a trend to improve on two dimensions of the Wellbeing Scale (*Affection* and *Assertivity*). The trained participants also improved in attention, showing a reduction in distraction and an increase of alertness at posttest compared to pretest. In contrast, visuospatial working memory and executive control functions did not improve immediately after training. These findings indicated the existence of significant transfer effects to untrained functions and an improvement of subjective wellbeing, suggesting a generalization of cognitive training beyond the trained non-action video games just after ending the training program. However, the usefulness of the intervention depends on two criteria: (a) transfer from the trained ability to abilities that were not trained; and (b) durability of the training effects.

In the present article we consider the durability of the effects of the training program. We report below the results of a 3-month follow-up assessment of the trained and control older participants in a randomized control trial after a no-contact period. The main goal was to find out whether the benefits of the intervention found at posttest in the trained group, as compared to the control group on several cognitive and wellbeing outcomes, would still be present after 3 months of no practice.

## Materials and methods

In this section, we summarize the methods used in this clinical trial. A detailed description of the methods and procedures used has been reported in two previous publications (Ballesteros et al., 2014; Mayas et al., 2014). The study was approved and conducted in compliance with the guidelines of the Ethical Review Board of the *Universidad Nacional de Educación a Distancia* (UNED).

### Participants

Forty healthy older volunteers (age range 57- to 80-years-of-age) participated in the study. After signing their informed consent, they were randomly assigned to either the experimental group or the control group before being evaluated on the tests and laboratory tasks. Inclusion criteria for the study were to: (1) have a score of 26 or above on the MMSE (Folstein et al., 1975); (2) obtain a score of under 5 on the Yesavage Depression Scale (Yesavage et al., 1983; Spanish adaptation by Martínez et al., 2002); (3) obtain a normal score on the Vocabulary subscale of the WAIS-III scale (Wechsler, 1999); (4) live an active independent life; (5) have normal or corrected-to-normal hearing and vision; and (6) not suffer from neurological, psychiatric disorders or traumatic brain injury. *T*-tests showed no significant differences between the two groups (all *ps* > 0.05) for all of these measures prior to the intervention. Table 1 summarizes the demographic information and screening test scores for the trained and control groups.

**Table 1:** **Demographic information for participants in each group**.

**Characteristic**	**Experimental**	**Control**	**η^2^*_p_***	***p***	***F***
Women/men (n)	10/7	8/5	0.001	0.885	0.021
Age (years)	68, 8 (5, 15)	69, 2 (5.91)	0.001	0.849	0.037
Education (years)	12.2 (5.09)	12.9 (3.28)	0.008	0.649	0.212
MMSE	28.7 (1.16)	28.8 (1.03)	0.001	0.847	0.038
Depression	1.5 (1.18)	2.4 (2.88)	0.035	0.320	1.023
Verbal ability	62.4 (9.43)	60.8 (7.37)	0.017	0.495	0.479

*Note. Means and Standard deviations (SDs in parentheses); MMSE, Mini-Mental State Examination, Verbal ability, Vocabulary (Wechsler); Depression, Yesavage Depression Scale*.

**Figure 1:**
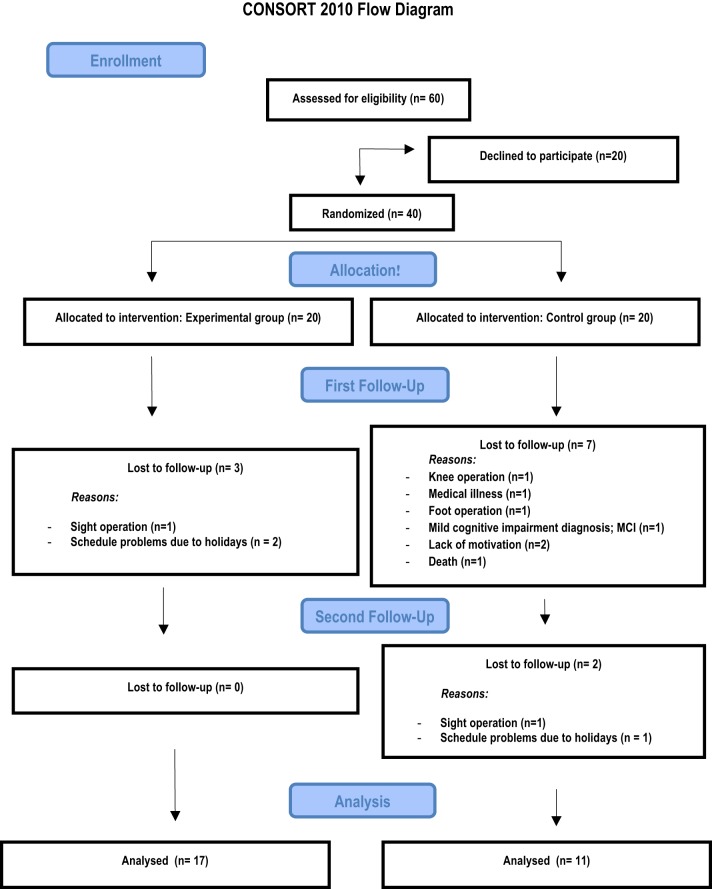
**CONSORT flowchart with the follow-up information**.

The training phase was completed by 17 of the 20 participants in the video game training group (1 dropout suffered from an eye operation during training and the other 2 dropouts had availability problems) and by 13 of the 20 participants in the control group (1 dropout had a knee operation, 1 had a foot operation, 1 died, 1 was diagnosed with MCI during the course of the study, and 2 were not motivated to continue). The follow-up assessment was completed by the 17 participants of the trained group and by 11 participants in the control group (one was missed due to an operation and another due to family holidays). Analyses of background characteristics revealed no differences between participants remaining in the study and the dropouts within the respective group, except in the Mini-Mental State Examination and this was due to a control participant diagnosed with MCI during the course of the study (see Figure 1).

### Design

The design was a single-blind randomized controlled study. After signing an informed consent form, participants were assigned randomly to the trained (experimental) group or to the control group. All participants were assessed on a series of cognitive tasks, tests and questionnaires described briefly below at 3 time points: pretest (baseline), posttest (after completing the training sessions or a similar time), and after a 3-month follow-up no-contact period.

Participants in the experimental and control groups attended individually 7 assessment sessions (screening, 2 pretest, 2 posttest, and 2 3-month follow-up sessions) in which they performed a series of laboratory designed experimental tasks (a cross-modal oddball task, a simple and a choice RT task, several spatial working memory tasks (Rey-Osterrieth Figure Test, Corsi Blocks, and Jigsaw-Puzzle Test), and the computerized Wisconsin Card Sorting Task (WCST) to assess possible transfer effects of training to different cognitive processes. Immediate and delayed episodic visual memory was assessed with the Wechsler Memory Scale-WMS-III (Memory Faces I and I, and Family Pictures I and II). Subjective wellbeing was also assessed with the IPF-IL scale (Nieboer et al., 2005).

### Intervention study

Participants assigned to the trained group attended 20 1-h training sessions over 10–12 weeks. In each training session, each participant practiced twice 10 video games selected from *Lumosity* (http://www.lumosity.com). The games were designed to improving the user's cognitive abilities (Stemberg et al., 2013). Transfer of training was measured as performance improvement at posttest relative to pretest and from pretest (baseline) relative to 3-month follow-up.

The experimental group practiced the 10 selected video games in our laboratory on a PC equipped with a 21-inch monitor (see Ballesteros et al., 2014, for a short description of the video games). These video games were *Speed mach, Memory matrix, Rotation matrix, Face memory, Memory match, Moneycomb, Lost in migration, Space junk, Raindrops, and Chalkboard*. Scores on each game were recorded. Participants received points based on their performance on each video game. In four games (*Memory match, Memory faces, Speed match, and Lost in migration*) the time necessary to complete the game was also recorded. The control group met three times with the researchers in a room of the laboratory. Each meeting lasted about 2 h. During these meetings members of the research group discussed with the participants general topics related to aging and their interests.

### Assessment tasks and procedures

Assessment measures included processing speed, attention, executive control, spatial working memory, episodic memory, and subjective wellbeing. The computerized tasks (simple and choice reaction time, oddball, Corsi blocks, and Jigsaw-puzzles) were programmed using E-Prime 2.0 (Psychology Software Tools Inc., Pittsburgh, PA, USA). Executive control was assessed with a computerized version of the Wisconsin Card Sorting Test (WCST). To perform these tasks, participants were comfortably seated at a distance of approximately 55 cm from the computer screen. The tasks and psychological tests are described briefly below.

### Cross-modal oddball attention task

Distraction and alertness were assessed with a cross-modal visual-auditory oddball task. Participants categorized a visual digit from 1 to 8 presented at the center of the computer screen as odd or even by pressing one of two keys (counterbalanced across participants). There were 3 blocks of 384 trials each. The sound conditions were three: A silent block and two block of trials containing two different sounds, the standard sound (used in 80% of the trials) that was a 600 Hz sine wave tone of 200 ms, and the novel sound (used in 20% of the trials; e.g., drill, hammer, rain).

### Speed of processing (simple and choice RT) tasks

Participants performed the simple and choice RT tasks in a counterbalanced order. In the simple RT task, participants viewed a target that appeared at the center of the computer screen and pressed a designated key as quickly as possible. In the choice RT task the stimuli “X” or “O” was presented and participants pressed a designated key for each of them as quickly as possible. Response keys were counterbalanced across participants. The dependent variable was response time for correct responses.

### Executive control (WCST)

Executive control was assessed with the WCST (CV4; Heaton, 1981). On each trial, four cards differing in shape (square, triangle, circle, or star), color (blue, red, yellow, or green) and number of shapes (one, two, three, or four) were displayed on the computer screen. The participant sorted the cards into different categories according to shape (S), color (C), or number (N) of shapes. The computer recorded each response as correct or incorrect. The task ended after 10 consecutive responses to each category in the order CSNCSN, or when the two sets of 64 response cards had been presented. The main dependent variable was the number of perseverative errors, which is associated with the shrinking of frontal areas (Gunning-Dixon and Raz, 2003).

### Visuospatial working memory (WM)

Spatial WM (Baddeley and Hitch, 1974) was assessed with the *Corsi blocks* and the *Jigsaw-puzzle tasks*, and the *Rey-Osterrieth Complex Figure Test*. Participants performed a computerized version of the *Corsi Task* with four difficulty levels (2, 3, 4, and 5 cubes). The stimuli appeared one by one on the computer screen inside a 10 × 10 cm matrix for 1000 ms each. On each trial, the participant reproduced the pattern of cubes just presented. The score was the proportion of correct sequences for each level. We used a computerized version of the *Jigsaw-Puzzle Task*. Richardson and Vecchi (2002) developed the pencil and paper task as an instrument to assess active visuospatial abilities. Our participants were presented with puzzles consisting of 4, 6, or 9 pieces on the computer screen. Each piece was numbered and the participant had to write down the number corresponding to the pieces in their correct spatial positions. In each trial, a fragmented picture appeared on the screen and the participant wrote down on the response sheet the appropriate numbers to form a spatially correct picture. The proportion of correct puzzles per level (4, 6, and 9 pieces) was the dependent variable. Participants also performed the Spanish adaptation of the *Rey-Osterrieth Complex Figure Test* (Rey, 1942, 1999), to assess visual constructive abilities and visuospatial memory. They reproduced a complex drawing, first by copying it and then by reproducing the drawing from memory.

### Immediate and delayed visual episodic memory

Immediate recognition memory for Faces and Family Pictures was assessed with Faces I, and Family Pictures I, while delayed memory (25 min after encoding) was assessed with Faces II and Family Pictures II, from the Spanish version (Wechsler and Pereña, 2004) of the Memory Wechsler Scale, WMS-III (Wechsler, 1997).

### Wellbeing

Subjective wellbeing was assessed with the 15-item short version of the SPF-IL Scale (Nieboer et al., 2005). Wellbeing is the overall state of comfort of a person determined by his/her ability to obtain the goals of physical and social wellbeing. The scale assesses 5 dimensions. The *Affection* dimension conveys the degree of confidence, social acceptance and level of satisfaction with the people around. *Assertivity* refers to the self-perception of having done the right thing in the eyes of relevant others. *Status* assesses the feeling of being treated with respect, self-realization, achievement as compared to others, and reputation. *Comfort* is the absence of feelings of discomfort, pain or stress. Finally, *Stimulation* refers to mental and physical activation.

## Results

The main questions examined in our randomized controlled study were whether training with non-action video games enhanced cognition of older adults and if training would transfer to other untrained broader cognitive functions. The questions were tested by considering whether group (control group, video game trained group) interacted with testing session (pre, post-training, 3-month follow-up) with regard to performance on a series of outcome cognitive measures. The specific question addressed in this phase of the study was whether the post-training benefits would still be present after 3 months without practice.

### Effects of the intervention on cognitive performance (post-training results)

As previously reported, video game performance improved significantly across training sessions, suggesting that training session was a reliable predictor of video game score and response time in all games. More important, however, were the effects of the intervention program on cognitive performance. The post-training results indicated that the trained group showed enhancements compared to control participants in several cognitive outcomes, including: (1) controlled processing, as shown by the significant improvement in the choice RT task; (2) attention, as the trainees were less distracted by irrelevant sounds and showed an increase in alertness; (3) immediate and delayed recall memory for family pictures (WMS-III); and (4) *Affection and Assertiveness* subscales of the Wellbeing scale. However, the trained group did not show transfer to executive control assessed with the WCST nor to spatial WM assessed with three different tasks. The post-training results were previously described in detail elsewhere (Ballesteros et al., 2014; Mayas et al., 2014).

### Maintenance of training at the 3-month follow-up

Table 2 displays pretest, posttest, and 3-month follow-up performance on the psychological measures and wellbeing dimensions for the trained and control groups. The main question examined in the follow-up part of this study was whether the enhancements of the cognitive functions found in the trained group at post-training remain at the 3-month follow-up. To answer this question, we conducted mixed analyses of variance (ANOVAs) with group (experimental, control) as the between-subjects factor and session (pre-training, post-training and 3-month follow-up) as the within-subjects factor. Analyses were conducted using SPSS software version 21 (IBM Corp. 2007-2012).

**Table 2:** **Pre-training, post-training, and 3-month follow-up performance on psychological measures and well-being dimensions for the experimental and control groups**.

		**EXP—CNTRL (DT_EXP/DT_CNTRL)**		
		**Pre-test**	**Post-test**	**3-Month follow-up**
Speed of processing (Less is better)	Detection (ms)	−6 (74/69)	+19 (76/75)	+39 (74/61)
	Choice RT (ms)	+26 (106/62)	−12 (80/86)	+49 (92/71)
Crossmodal oddball task	Distraction (ms) (less is better)	+1.5 (34/31)	−9.9 (25.5/27.7)^*^	+3.9 (26.5/26.8)
	Alertness (ms)	−17.9 (25/33)	+15 (35.8/45.5)^*^	+15.1 (33.7/46.2)
WCST	Error (%)	−5 (15.5/17.9)	−1.1 (18.2/16.2)	−2.6 (15.9/17.1)
	Perseverative Resp (%)	+0.2 (12.6/9.7)	+2.6 (14.3/8.3)	−0.5 (11.4/8.9)
	Perseverative Error (%)	−0.7 (9.3/8.7)	+1.7 (11.3/7.0)	−0.7 (9.1/8.4)
	Non-Persev. Error (%)	−4.3 (8.5/8.3)	−3.2 (12.8/12.9)	−2.2 (8.2/9.3)
	Conceptual Level (%)	+5.8 (23.1/25.3)	+3.2 (23.6/22.1)	+3.7 (23.9/25.2)
Jigsaw Puzzle task	4 Pieces (proportion)	0 (0.26/0.22)	+0.12 (0.28/0.28)	−0.03 (0.30/.25)
	6 Pieces (proportion)	+.05 (0.25/0.33)	+.09 (0.48/0.28)	+0.07 (0.30/0.29)
	9 Pieces (proportion)	−0.04 (0.05/0.09)	−0.07 (0.17/0.21)	−0.04 (0.17/0.28)
Corsi Blocks task	2 Serial Position (proportion)	+0.01 (0.07/0.07)	0 (0.05/0.04)	+0.06 (0.02/0.10)
	3 Serial Position (proportion)	+0.16 (0.16/0.26)	−0.05 (0.25/0.13)	0 (0.10/0.15)
	4 Serial Position (proportion)	+0.19 (0.20/0.21)	+0.19 (0.27/0.25)	+0.14 (0.21/0.21)
	5 Serial Position (proportion)	+0.04 (0.22/0.24)	+0.14 (0.23/0.17)	+0.04 (0.18/0.18)
Rey	Copy	+1.7 (3.1/3.3)	−1.2 (4.6/8.7)	−0.9 (2.6/1.3)
	Delayed Recall	+0.3 (6.6/3.5)	−1.1 (6.6/7.2)	−3.1 (7.1/8.5)
WMS-III	Faces (Immediate)	−0.9 (4.7/4.0)	−0.03 (4.5/4.0)	+0.3 (6.0/6.7)
	Faces (Delayed)	−1.5 (4.1/3.4)	−0.9 (5.2/5.6)	−1.2 (6.5/4.8)
	Family Pictures (Immediate)	+0.6 (6.3/7.3)	+6.3 (8.1/9.6)^*^	+4.4 (9.2/9.5)
	Family Pictures (Delayed)	+1 (6.5/6.0)	+7.8 (8.5/9.1)^*^	+6.6 (10.7/7.5)
SPF-IL	Affect	−0.5 (1.8/2.3)	+0.8 (1.7/1.7)^*^	1 (1.6/1.5)^*^
	Assertiveness	−0.2 (1.7/1.1)	+0.8 (1.3/1.4)^**^	+1.8 (1.3/1.5)
	Status	−0.4 (1.8/1.2)	+0.6 (1.7/1.8)	+0.7 (1.4/1.8)
	Comfort	−0.3 (1.5/1.5)	−0.2 (2.1/1.9)	0 (1.4/2.3)
	Stimulation	+1 (1.3/1.1)	+1 (1.6/1.7)	+0.7 (1.5/1.2)

*Note: EXP, Experimental group; CNTRL, Control group; SD, Standard deviation; Differential scores (experimental minus control of the outcome measures. Positive scores means better performance of the experimental group compared to the control group (except for RTs and distraction measures where negative scores mean better performance of the experimental group). WCST (Wisconsin Card Sorting Test); REY (Rey-Osterrieth Complex Figure Test); WMS-III (Wechsler Memory Scale); Dimensions of Wellbeing (SPF-IL Scale)*;

** *indicates tests on which the experimental group showed significant greatly improvements than controls (p < 0.05)*;

* *indicates the test on which there was a trend for larger improvements in the experimental group (p < 0.10); + indicates that the control group showed significantly greater improvements than the experimental group (p < 0.05)*.

### Cross-modal oddball attention task

**Figure 2:**
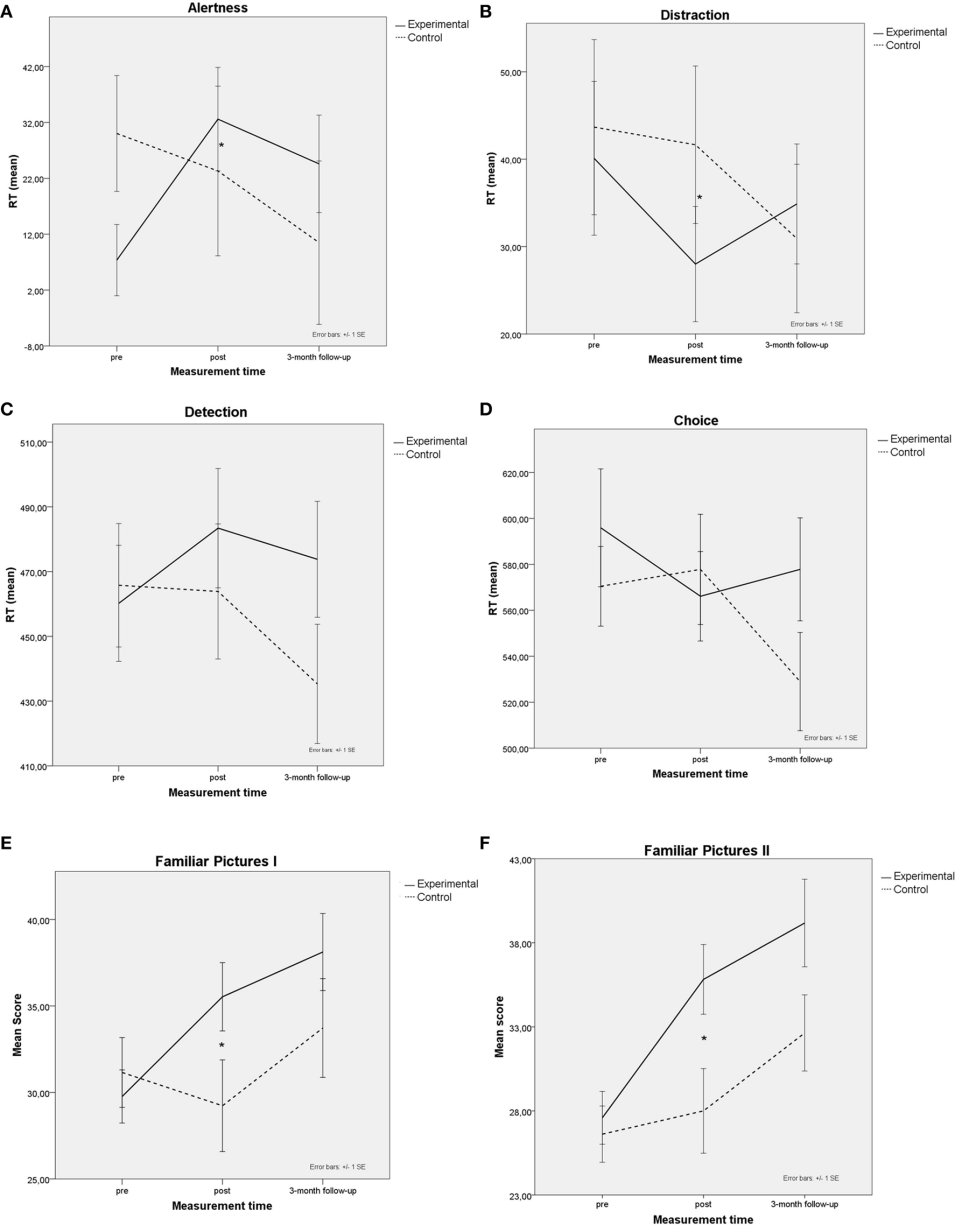
**Mean performance of trained (solid lines) and control groups (dashed lines) at pretest, posttest and 3-month follow-up. (A)** Distraction effects in the Cross-modal oddball attention task. **(B)** Alertness effects in the attention task. **(C)** Simple reaction time task. **(D)** Choice reaction time task. **(E)** Family pictures I, Immediate. **(F)** Family pictures II, Delayed. Bars represent standard error of the mean (SE); * *p* < 0.05.

We used a cross-modal visual-auditory oddball task programmed using E-prime 2.0 (Psychology Software Tools Inc, Pittsburg, PA, USA) to assess distraction and alertness (see Mayas et al., 2014, for more details). After completing the training (post-test) the experimental group showed a significant improvement in the level of alertness and a reduction in distraction. However, these benefits disappeared at the 3-month follow-up for attentional effects, alertness [*F*_(2, 46)_ = 2.27, *MSE* = 2395.1, *p* > 0.05, η^2^_*p*_ = 0.08] and distraction [*F*_(2, 46)_ = 1.98, *MSE* = 468.2, *p* > 0.05, η^2^_*p*_ = 0.09]. See, Figures 2A,B.

### Simple and choice RT tasks

Speed of processing was evaluated with simple and choice RT tasks programmed using E-Prime 2.0. In the simple RT task, participants responded as quickly as possible to a target (the letter “X”) that appeared at the center of the computer screen. In the choice RT task participants responded to different stimuli (the letter “X” or the letter “O”) pressing a designated key for each of them. Response keys were counterbalanced across participants (see Ballesteros et al., 2014, for more details). After completing the training (posttest), the experimental group showed a significant improvement in the choice RT task compared with the control group, but this disappeared at the 3-month follow-up. The three-way interaction group x task x assessment was not significant [*F*_(2, 52)_ = 2.02, *MSE* = 1413, 31, *p* > 0.05, η^2^_*p*_ = 0.07]. Only the main effect of task was significant [*F*_(1, 26)_ = 3443.69, *MSE* = 1413, 31, *p* > 0.05, η^2^_*p*_ = 0.83], showing that both groups were faster in the simple RT task compared with the choice RT task. See Figures 2C,D.

### Visual episodic immediate and delayed recall

Immediate and delayed recall memory was assessed using Family Pictures I and II subscales from Wechsler Memory Scale, WMS-III (Wechsler, 1997). After completing the training (posttest measure) *post-hoc* comparisons showed that the experimental group had a significant improvement in the immediate and delayed recall scores, but this improvement disappeared at the 3-month follow-up [*F*_(1, 26)_ = 2.35, *MSE* = 199.48, *p* > 0.05, η^2^_*p*_ = 0.08]. See Figures 2E,F.

### Wellbeing

**Figure 3:**
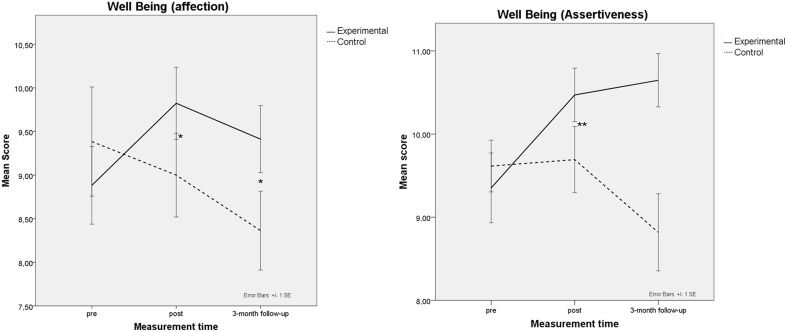
**Mean performance of trained (solid lines) and control groups (dashed lines) at pretest, posttest and 3-month follow-up in subjective wellbeing: (Right) *Assertivity*; (Left) *Affection***. Bars represent standard error of the mean (SE); * *p* < 0.05; ** *p* < 0.001.

Subjective wellbeing was assessed with the short version of the SPF-IL Scale (Nieboer et al., 2005), which evaluates five different wellbeing dimensions. The previous marginally significant interaction group x session between experimental and control groups on the *Affection* subscale [*F*_(1, 26)_ = 3.04, *MSE* = 7.34, *p* = 0.09, η^2^_*p*_ = 0.09], and on the *Assertiveness* subscale remained marginally significant [*F*_(1, 26)_ = 11.27, *MSE* = 22.34, *p* < 0.01, η^2^_*p*_ = 0.30] at the follow-up, as evidenced by the pair comparisons conducted (see Figure 3 Left and Right).

Participants also completed two visuospatial working memory tasks (Jigsaw and Corsi blocks) and an executive control (shifting strategy) task, the Wisconsin Card Sorting Test (WCST). There was no significant difference between experimental and control groups on these tasks (*p* > 0.05) after completing the training (posttest) and the same results were found again at the 3-month follow-up assessment (*p* > 0.05).

## Discussion

The goal of the present phase of this intervention study was to assess the stability of a series of positive cognitive outcomes of training with non-action video games after a 3-month no-contact follow-up period. The analyses performed on the cognitive outcomes at posttest had yielded two main findings. Firstly, the results indicated that the trained group showed enhancements compared to the control group in controlled processing, as shown by the significant improvement in the choice RT task; in attention, as the trainees were less distracted by irrelevant sounds and showed an increase in alertness; in immediate and delayed recall memory for Family Pictures (WMS-III) as well as in *Affection and Assertiveness* of the Wellbeing scale. Given the age-related declines in these cognitive functions, the results were promising. Secondly, however, our trained participants neither showed transfer to executive control assessed with the WCST nor to spatial WM assessed with three different tasks at posttest compared to pretest.

In the present phase of this clinical trail, we evaluated the maintenance of cognitive benefits after a no-contact period. Our results showed that most of the benefits obtained after training vanished at the 3-month follow-up. These findings are in agreement with results from Stern et al. (2011). These authors investigated the effect of training cognitively healthy older adults with Space Fortress (a complex video game) to improve executive control. Their study consisted of 36 1-h video game training sessions over 3 months with cognitive evaluations before, after, and at a 3-month follow-up. The results showed modest transfer to just one measure of executive control (the letter-number sequencing task) at posttest in the experimental group but the benefit vanished after 3 months of no game playing.

Buschkuehl et al. (2008) conducted an adaptive visual working memory (WM) training intervention with old-old adults (mean age = 80 years). The results showed substantial gains in the trained task and improvements immediately after training completion in visual WM that disappeared at the 1-year follow-up. Contrasting with these results, Anguera et al. (2013) reported reduced multitasking costs on the trained group compared to both an active control group and a passive control group. After training, older adults performed better than untrained 20-years adults, and these gains persisted after 6 months. Anguera et al. (2013) trained older adults in multitasking performance using an adaptive version of a custom-designed three-dimensional video game (NeuroRacer).

Other training studies conducted with cognitively normal older adults found cognitive improvements and maintenance effects over time of trained cognitive abilities as well as transfer to activities of daily living (Ball et al., 2002; Willis et al., 2006; Cheng et al., 2012; Mitchell et al., 2012). Mahncke et al. (2006) used a computer-based cognitive program for 8–10 weeks in 3 groups, an experimental group, an active control group and a no-contact control group. They found improved verbal memory performance immediately after training and generalization to untrained tasks. Short-term memory improvements remained at the 3-month follow-up in the experimental group. Overall, evidence for maintenance cognitive training effects is available but limited and needs further investigation.

Valenzuela and Sachdev (2009) conducted a meta-analytic study to examine transfer effects of training to untrained domains, and the persistence of the benefits over a follow-up period, after training in 7 randomized controlled trials (RCT). The results of the meta-analysis showed a moderate effect size of 0.6 on average. Cognitive training of healthy older adults improved performance on the trained task immediately after the end of the intervention. The meta-analysis also showed that 2–3 months of training have long-lasting effects on cognition in cognitively normal older adults (between 1.2 and 2.6 points in the MMSE). Although promising, these results must be interpreted with caution because only the primary outcome variable per clinical trial was included in the meta-analysis, while secondary outcomes (usually less robust) were not included.

Our results as well as other follow-up findings after training can be explained by the *Scaffolding Theory of Aging and Cognition “STAC”* (Park and Reuter-Lorenz, 2009), and especially by the revised model “*STAC-r*” (Reuter-Lorenz and Park, 2014). This theory suggests that the human brain adapts and reorganizes with new learning and cognitive training, improving the ability to scaffold and develop new neural circuitry to compensate for age-related decline (Goh and Park, 2009). Importantly, the construct “*neural resource enrichment*” introduced in STAC-r allows for positive influences to the brain. Life course enrichment factors could increase compensatory scaffolding protecting the older brain against cognitive decline. Both theories include the possible benefits of structured interventions to enhance compensatory scaffolding, improving cognitive functioning in aged individuals. There are similarities between certain aspects of the *cognitive and brain reserve theory* (Stern, 2002; Barulli and Stern, 2013), the approach taken by Lövdén et al. (2010), and the STAC-r theory. Lövdén et al. (2010) suggest that when people are faced with a prolonged mismatch between functional supply and (intrinsic or extrinsic) challenge, their brains exhibit plastic and compensatory changes. Enriching variables (e.g., new learning, social engagement, exercise, cognitive training) as well as depleting variables (e.g., stress, depression, head trauma, toxin exposure) cited in STAC-r can enhance or diminish cognitive and brain reserve. It seems very likely that video game training supports general scaffolding, but this is too fragile to remain assembled over time, with only some of its parts being resistant to decline after training is over.

Future research is needed to overcome some limitations of video game and cognitive training studies. Among these limitations are (a) the relatively small sample sizes of most longitudinal intervention studies (e.g., Clark et al., 1987; Goldstein et al., 1997; Cassavaugh and Kramer, 2009; Stern et al., 2011; Maillot et al., 2012; Nouchi et al., 2012; Ballesteros et al., 2014); and (b) the difficulty of ruling out effects due to motivational factors (Boot et al., 2013b) and familiarity with the investigators (the so-called *placebo effect*) as most studies included just a passive, no-contact control group (e.g., Goldstein et al., 1997; Basak et al., 2008; Maillot et al., 2012; McDougall and House, 2012; Ballesteros et al., 2014) or no control group (Cassavaugh and Kramer, 2009; Ackerman et al., 2010). However, it is worth mentioning that Toril et al. (2014) calculated the effect sizes of those studies that used both an active and a passive control group (5 out of the 20 published studies included in the meta-analysis). The mean effect size (Cohen's *d*) for active control was 0.36 and for passive control 0.37. Another possible limitation of the present study is the lack of inclusion of groups of young adults. It is known that young adults benefit more from training than older adults (Dahlin et al., 2008) as plasticity decreases with aging (Brehmer et al., 2007). The inclusion of young adults would inform on the effect of age. It is possible that the benefits of training that fade over time in older adults remain in younger adults.

Future directions for research include developing custom-designed video games to improve specific cognitive functions, as a way to create more powerful tools for cognitive improvement not only for cognitively healthy older adults but also for individuals with mild cognitive impairment.

The current study had relatively small sample sizes as have other recent randomized training studies (e.g., Mozolic et al., 2011; Hampstead et al., 2012; Nouchi et al., 2012). Despite that, we found at posttest that participants who completed the 20 training sessions were faster on a choice RT task, showed reduced distraction and an increase in alertness on an oddball attention task, improved memory for visual stimuli and improved (marginally) in two dimensions of subjective wellbeing just after training. These results suggest that the brains of the older adults retain some neurocognitive plasticity and benefit from scaffolding just after training. However, cognitive enhancements are too fragile to persist over time without mental practice.

Another limitation of the present study is related to the difficulty of ruling out possible effects due to motivational factors, familiarity or confidence with the researchers rather than neurocognitive plasticity, as the trained group had more contact with the experimenters than the control group. Hence a characteristic of our future research will be to include two types of control groups, an active control group and a no-contact control group. While recognizing these limitations, this study comprises a first step to assess the usefulness of training older adults with non-action video games, which seem more appropriate for older adults than other genres of video games.

## Summary and conclusions

The results of our randomized controlled study suggest that training older adults with non-action video games enhances important aspects of cognition including attention, processing speed, and memory as well as subjective wellbeing (*Assertiveness* and *Affection* dimensions) just after the completion of the training program. However, except for two wellbeing dimensions that remained marginally significant, these cognitive enhancements vanished after 3 months of no game play. Transfer effects were not maintained over these 3 months for attention (distraction and alertness), processing speed (assessed by a choice reaction time task), and visual memory (immediate and delayed). This result suggests that, as occurs in other interventions, to maintain training improvements it may be necessary to continue practicing the games from time to time.

Overall, the present results are consistent with the idea that structured interventions enhance compensatory scaffolding (Reuter-Lorenz and Park, 2014), supporting at least some cognitive functions as predicted by the STAC-r theory and the reserve hypothesis (Stern, 2002, 2009, 2012). Cognitive plasticity can be induced in older adults by training. However, to maintain the benefits provided by the training intervention some boosting sessions would be necessary.

The inconsistencies reported in the field of video game cognitive training do not allow us to reach a very robust conclusion regarding the potential of video game training interventions for older adults (see Karbach, 2014). The key topic for future research is to find out how to design training regimes that produce lasting training benefits in the older mind and brain.

## Author note

The authors would like to inform that they have not had any contact with the package manufacturers of the *Lumosity* cognitive training platform at any time during the duration of the study. This study was conducted independently from them.

### Conflict of interest statement

The authors declare that the research was conducted in the absence of any commercial or financial relationships that could be construed as a potential conflict of interest.
